# Nucleic Acid Sensors Involved in the Recognition of HBV in the Liver–Specific *in vivo* Transfection Mouse Models—Pattern Recognition Receptors and Sensors for HBV

**DOI:** 10.3390/medsci3020016

**Published:** 2015-04-15

**Authors:** Chean Ring Leong, Hiroyuki Oshiumi, Takayuki Suzuki, Misako Matsumoto, Tsukasa Seya

**Affiliations:** Department of Microbiology and Immunology, Hokkaido University Graduate School of Medicine, Kita 15, Nishi 7, Kita-ku, Sapporo 060-8638, Japan; E-Mails: crleong@med.hokudai.ac.jp (C.R.L.); oshiumi@med.hokudai.ac.jp (H.O.); kai_dirk.big-violet@med.hokudai.ac.jp (T.S.); matumoto@pop.med.hokudai.ac.jp (M.M.)

**Keywords:** RNA sensors, DNA sensors, hepatitis B virus, knockout mouse

## Abstract

Cellular innate immune system recognizing pathogen infection is critical for the host defense against viruses. Hepatitis B virus (HBV) is a DNA virus with a unique life cycle whereby the DNA and RNA intermediates present at different phases. However, it is still unclear whether the viral DNA or RNA templates are recognized by the pattern-recognition receptors (PRRs) to trigger host antiviral immune response. Here in this article, we review the recent advances in the progress of the HBV studies, focusing on the nucleic acid sensors and the pathways involved in the recognition of HBV in the liver–specific *in vivo* transfection mouse models. Hydrodynamic injection transfecting the hepatocytes in the gene-disrupted mouse model with the HBV replicative genome DNA has revealed that IFNAR and IRF3/7 are indispensable in HBV eradication in the mice liver but not the RNA sensing pathways. Interestingly, accumulating evidence of the recent studies has demonstrated that HBV markedly interfered with IFN-β induction and antiviral immunity mediated by the Stimulator of interferon genes (STING), which has been identified as a central factor in foreign DNA recognition and antiviral innate immunity. This review will present the current understanding of innate immunity in HBV infection and of the challenges for clearing of the HBV infection.

## 1. Introduction

Hepatitis B virus (HBV) is one of the significant health burdens worldwide as more than 240 million people are chronically infected [1]. HBV infections in the adults have a relatively low rate of chronicity (about 5%) while neonatal infections usually have a high persistence rate [2]. Although the underlying mechanisms by which HBV establishes and maintains chronic infection are poorly understood, it is generally believed that the consequence of HBV infection is a result of a complex interaction between the host immune system and replicating virus [3].

The role of the adaptive immune responses in the clearance of HBV infection is widely accepted. However, the role of innate immune response and capacity of HBV to induce innate responses remain controversial. Type I interferon (IFN), a representative output of antiviral innate signaling, seems undetectable or missed in most cases of HBV patients [4]. Thus far, we have no concrete evidence that IFNs are produced in HBV patients. The immune responses to HBV in various phases of patients appear difficult to understand, largely due to the narrow host restriction and complex life-cycle of this pathogen and the limitation of existing experimental models, including human cell-culture system and animal models. Experimental viral infection in both chimpanzees and woodchucks found only limited or even non-activation of innate immunity being demonstrated in acute HBV infection [5], although some technical problems may have disturbed the detection of IFN.

Nevertheless, some recent studies suggested that in the early phases of infection HBV could activate some of pattern-recognition receptors (PRRs), which in turn limited the infection and drove adaptive immune responses crucial to the virus clearance [6,7]. For instance, HBV replication in HepRG (a human hepatocyte) cell lines was shown leading to an early activation of the innate immune response [6]. Hence, it is vital to analyze how the innate immune response to HBV infection is driven by a specific “pathogen-associated molecular pattern (PAMP)”. However, the mechanisms accountable for HBV sensing have not been clarified and the question of whether the HBV DNA or RNA templates are the PAMPs that trigger the antiviral response remains unresolved. Here we are summarizing our mouse-model study on the recognition system for HBV nucleic acid PAMP in the host innate immune system as well as the innate immune signaling pathway of varies nucleic acid sensor interfered by the HBV viral proteins with reference to recent findings in literatures (Table 1).

**Table 1:** Innate immune signaling pathway of varies nucleic acid sensor interfered by the HBV viral proteins.

Nucleic Acid Sensor Related Cellular Targets	HBV Viral Proteins	References
TLR2-pathway	HBs, HBe	(Wang, S., *et al*. 2013) [8]
TLR3-pathway	Polymerase	(Yu, S., *et al*. 2010) [9]
TLR4-pathway	HBs	(Cheng, J., *et al*. 2005) [10]
TLR9-pathway	HBs	(Vincent, I.E., *et al*. 2011; Xu, Y., *et al*. 2009) [11,12]
RIG-I-pathway	Polymerase, HBx	(Wang, H., *et al*. 2010; Kumar, M., *et al.* 2011) [9,13]
MDA5-pathway	HBx	(Wei, C., *et al*. 2010) [14]
STING-pathway	Polymerase	(Liu, Y., *et al*. 2015) [15]

## 2. RNA Sensors for Detection of HBV Infection

Viral RNA is sensed by the innate immune system via either Toll-like receptor 3 (TLR3) or cytoplasmic sensors such as retinoic acid-inducible gene-I (RIG-I) and melanoma differentiation-associated gene 5 (MDA5). RIG-I and MDA5 mainly participate in type I interferon (IFN) induction in conjunction with the adaptor molecule, mitochondrial antiviral signaling protein (MAVS; also called IPS-1, Cardif, or VISA). The Toll/IL-1R homology domain-containing adaptor molecule 1 (TICAM-1; also called TRIF) is the adaptor of TLR3, which senses viral RNA on the endosomal membrane. A few reports have suggested that the antiviral response against HBV is mediated by the RIG-I/MAVS pathway in the cytosol and its activation is blocked by HBV polymerase in infected cells [9,16]. However, no definitive evidence *in vivo* is available because analysis on the gene expression and effectors required for elimination of the replicative template has been especially difficult. Therefore, it remains skeptical for the assumption that the HBV RNA is the PAMP and caution is necessary in the evaluation of the physiological role of this HBV-mediated IFN-inducing mechanism since the agonists PAMPs in these studies are originated from various viruses or synthetic double-stranded (ds) RNA (such as Poly I:C). In addition to this TLR3, RIG-I and MDA5, there are a number of cytoplasmic RNA sensors, including PKR, LRRFIP1, DEAD box helicase proteins (DDX1, DDX3, DHX9, DHX21, DHX36 and DDX60), some RLRs and dicer-associated RNA-binding proteins. It remains untested whether these RNA sensors are associated with detection of HBV RNA in human hepatocytes. Since viral clearance is a multifaceted process, the full spectrum of immunological requirements for HBV clearance is not completely defined with the current *in vitro* studies using hepatocytes derived cell lines. A mouse model was developed by Yang *et al.* to provide a better insight into the host and viral interaction, in which hydrodynamic injection of a naked plasmid DNA encoding a supergenomic HBV1.3-length transgene into inbred mice initiates high-titer HBV replication in the liver that is rapidly terminated if the mice are immunocompetent [17,18]. Using the hydrodynamic injection and gene-disrupted mouse system, we investigated the role of RNA sensing pathways in controlling the HBV replication. Surprisingly, similar self-limiting pathogenesis of HBV was observed in the mice lacking the comprehensive RNA-sensing pathway, *Mavs^−/−^*, *Ticam1^−/−^* or *Mavs^−/−^*/*Ticam1^−/−^* double KO mice [19]. On the other hand, impairment of the molecules essential to the type I IFN inducing pathways *Irf-3/7^−/−^* or the IFN-α/β receptor pathway, *Ifnar^−/−^*, led to a greatly elevated virus titers in the KO mice even though the intrahepatic DNA template was eventually eliminated [19]. These results indicate that the suppression of type I IFN induction is necessary to contain the propagation of HBV at the early stage; however the RNA-sensing pathways are dispensable in suppressing the HBV replication.

Interestingly, our attention has also been called to the fact that lacking the MyD88 adaptor molecule which participates in the TLR9 pathway leads to the failure of the mice to eliminate the HBV viral template [19]. MyD88 is a representative adaptor for TLRs and IL-1β receptor, which activation is a result of inflammasome activation where PAMP-sensing caspases and RLRs are involved. Such findings suggest that the HBV-sensing is more complex than expected and the growing family of PRRs able to discriminate intracellular pathogen DNA might be the answer.

## 3. DNA Sensors in HBV Sensing

In recent years, the cytosolic DNA sensors which signal through STING have been demonstrated to be essential in eliciting innate immune responses against foreign DNA [20]. Most recently, cGAS was shown to signal the cytokine induction by activating the endoplasmic reticulum resident STING via cGAMP as a secondary messenger [21]. The latest report has revealed that STING is a new target of the viral polymerase to antagonize the IFN induction against HBV [15] and STING agonists induce an innate antiviral immune response against HBV [22]. Such finding is within the realm of possibility as HBV is an incomplete dsDNA virus of 3.2 kb, and more importantly, there are at least two types of viral DNAs with unique nucleic acid features compared with the host DNA: the relaxed circular DNA (rcDNA) and covalently closed circular DNA (cccDNA) in the HBV life cycle [23]. It is known that the IFN-inducing pathways downstream of the cytoplasmic and endosomal RNA sensors converged upon TBK1, a kinase for activating IRF3 that is shared by the cGAS-STING axis [24]. The phosphorylated form of TBK1, named pTBK1 could reflect the activation of either the RNA- and/or DNA-sensing pathway [25]. Moreover, we found that the pTBK1 generation in the mouse hepatocytes was depended on the overexpressed STING pathway under the presence of HBV DNA, suggesting that at least in the mouse pattern-sensing system, STING senses HBV DNA that links to IFN-α/β induction [25]. However, it is clear that much additional information will be required to understand the mechanism how HBV DNA is recognized particularly using the animal models that permit the natural infection of HBV.

It may however, be noted that the endogenous expression level of the cGAS-STING axis is rather low in the hepatocyte-derived cell lines, particularly the commonly used Huh7 and HepG2 in comparison to the macrophages or dendritic cells [19]. This may also suggest that such DNA sensing pathways (*i.e*., cGAS and STING) may have a rather peripheral role in sensing HBV replication in the hepatocyte lines, and that host cells other than hepatocytes contribute to regulation of an HBV infectious state. However, such responses need to be validated in other infection systems and in primary human hepatocytes.

There have been reported many other cytoplasmic and membrane-associated DNA sensors [20], some of which may actually take part in HBV DNA recognition in several steps of HBV genome synthesis in nucleus and cytoplasm of hepatocytes. RIG-I also acts as a DNA sensor in some cases. Both RIG-I and STING may participate in HBV-mediated IFN-induction in human hepatocyte lines. Since HBV propagation involves DNA and RNA replication phases, HBV DNA/RNA recognition would be more complicated than expected.

## 4. Overviews and Perspectives on HBV Sensing

The liver is an immunological organ with non-parenchymal cells (Kupffer cells, sinusoidal endothelial cells) and parenchymal cells (hepatocytes and epithelial cells) with unique immunosuppressive characteristics [26]. Besides the hepatocytes, numerous innate immune cells reside in the livers. HBV was also shown to be recognized by those non-parenchymal cells, primarily Kupffer cells, which displayed an innate immune response to restrain the viral propagation [27]. Furthermore, non-parenchymal cells may as well be involved in the formation of infectious nest and microenvironment that facilitate persistent infection and inflammation in hepatitis [28]. In recent years, it has become clear that inflammatory microenvironment in the innate immunity plays a critical role in the development of a number of liver diseases, including hepatocellular carcinoma (HCC). In particular, the activation of Toll-like receptor signaling results in the generation of immune responses and often results in the production of pro-inflammatory cytokines and chemokines, and could lead to acute inflammation in the liver [28]. Although only rare patients have smolder inflammation secondary to HBV infection, accumulating evidence suggest the prolonged inflammation provides microenvironment that serves as a nest for carcinogenesis. Immune cells as well as HBV factors unequivocally participate in the formation of microenvironment. Therefore, it is crucial to examine the HBV infection with a higher degree of participation of other immune cells for the primary response to HBV infection.

The life cycle of HBV consisted of a replication strategy that is thought to facilitate the HBV evasion of innate immunity. During HBV infection, the HBV genome is delivered into the nucleus. Infection is defined by the formation of covalently closed circular DNA. Following formation of covalently closed circular DNA, viral mRNA and pregenomic RNA is transcribed, which are to be capped and polyadenylated by host machinery [29,30]. The transcripts are exported to the cytoplasm but the viral RNA-DNA chimeric replicative genome is sequestered within viral capsids. Thus it seems that HBV is almost invisible to the innate sensing machinery. However the most recent study of Sato *et al.*, shows that RIG-I can counteract the interaction of HBV polymerase (P protein) with the 5'-ε region of pgRNA in an RNA-binding dependent manner, resulting in the suppression of HBV replication using a HepG2 culture and a chimeric mice with humanized livers [31]. IFN-λ is a main output in response to RIG-I stimulation by HBV pgRNA [31]. The results might reflect an aspect of an HBV infection. Nevertheless, the HepG2 cell line system does not always represent the hepatic milieu with inflammation in HBV infection, and human hepatocyte chimeric mice are immunologically compromised, far from a sufficient model for HBV infection. In these models, participation of non-parenchymal cells with an appropriate activation state can be barely investigated. Thus, the model systems appear to be too short to evaluate the whole immune responses occurring in infectious foci of HBV, where the combination of parenchymal and non-parenchymal cells substantially take part in the inflammatory microenvironment.

Although these observations from mouse models reflect some direct and indirect ways of the host and viral interactions, the real situation in the virus-infected patients needs to be assessed and further validation in relevant infection models in the near future is required. Identification of entry receptors for HBV (including NTCP) will facilitate the analysis of HBV infection in animal models. Comprehensive screening of host proteins that interact with the HBV proteins will give us valuable hints for HBV persistent infection.

**Figure 1:**
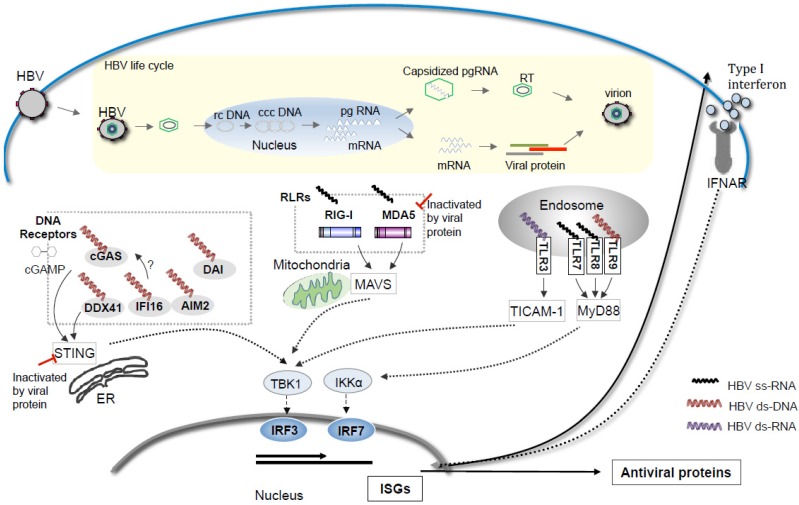
Possible Nucleic acid sensors participation in HBV pattern sensing in the life cycle of HBV. Various forms of nucleic acid are generated during the life cycle of HBV including the single stranded RNAs, double-stranded rc DNA/ccc DNA, or the double stranded RNAs. The cytosolic DNA receptors including cGAS, DDX41, IFI16, AIM2 and DAI may recognize the cytosolic DNA and activate the ER bound STING-TBK1-IRF3 axis to induce type I IFN production. While the RIG-I-like receptors (RLRs) including RIG-I and MDA5 activate the TBK1-IRF3 axis via mitochondria bound MAVS as adaptor protein upon the binding to cytosolic RNA. The four Toll-like receptor (TLR) family members: TLR3, TLR7/8 and TLR9 are located in the endosome, where they detect double stranded RNA, single-stranded RNA or non-methylated CpG DNA, respectively, leading to activation of TICAM-1 and MyD88 dependent pathways and the up-regulation of type I IFN. The HBV viral proteins seem to block the activation of the cytosolic RNA-sensing pathway as well as the cytosolic DNA-sensing pathways. Studies show that the HBV DNA could activate the ER bound STING and TBK1 in the hepatocytes and cytosolic RNA-sensing is dispensable in limiting the HBV propagation in the mice receiving liver specific *in vivo* transfection of the replicative HBV genome [19,25].

Meanwhile, increasing studies suggest that the growing family of PRRs able to discriminate intracellular pathogen DNA might be the answer to the nucleic acid sensor to HBV. DNA sensors may take a two-pronged attack on the life-cycle of HBV, while RNA sensors target viral RNA. It remains undetermined which is more implicated in the elimination of HBV. Our current knowledge on the nucleic acid-sensing pathways of HBV based on the mouse hydrodynamic studies is schematically summarized in Figure 1. While, the innate immune signaling pathway of various nucleic acid sensors and their regulation by the HBV viral proteins are summarized in Table 1.

## Abbreviations

cGAS: Cyclic GMP-AMP synthase
TLR3: Toll-like Receptor 3
MDA5: Melanoma Differentiation-Associated protein 5
RIG-I: retinoic acid-inducible gene-I
MyD88: Myeloid differentiation primary response gene (88)
MAVS/IPS1: Mitochondrial antiviral-signaling protein
TICAM-1: TIR domain containing adaptor molecule 1
IRF: interferon regulatory factor
STING: Stimulator of interferon genes

## References

[1] Dienstag J.L. (2008). Hepatitis B virus infection. N. Engl. J. Med..

[2] McMahon B.J. (2005). Epidemiology and natural history of hepatitis B. Semin. Liver Dis..

[3] Wieland S., Thimme R., Purcell R.H., Chisari F.V. (2004). Genomic analysis of the host response to hepatitis B virus infection. Proc. Natl. Acad. Sci. USA.

[4] Dunn C., Peppa D., Khanna P., Nebbia G., Jones M., Brendish N., Lascar R.M., Brown D., Gilson R.J., Tedder R.J. (2009). Temporal analysis of early immune responses in patients with acute hepatitis B virus infection. Gastroenterology.

[5] Bertoletti A., Gehring A.J. (2006). The immune response during hepatitis B virus infection. J. Gen. Virol..

[6] Lucifora J., Durantel D., Testoni B., Hantz O., Levrero M., Zoulim F. (2010). Control of hepatitis B virus replication by innate response of HepaRG cells. Hepatology.

[7] Shlomai A., Schwartz R.E., Ramanan V., Bhatta A., de Jong Y.P., Bhatia S.N., Rice C.M. (2004). Modeling host interactions with hepatitis B virus using primary and induced pluripotent stem cell-derived hepatocellular systems. Proc. Natl. Acad. Sci. USA.

[8] Wang S., Chen Z., Hu C., Qian F., Cheng Y., Wu M., Shi B., Chen J., Hu Y., Yuan Z. (2013). Hepatitis B virus surface antigen selectively inhibits TLR2 ligand-induced IL-12 production in monocytes/macrophages by interfering with JNK activation. J. Immunol..

[9] Yu S., Chen J., Wu M., Chen H., Kato N., Yuan Z. (2010). Hepatitis B virus polymerase inhibits RIG-I- and Toll-like receptor 3-mediated beta interferon induction in human hepatocytes through interference with interferon regulatory factor 3 activation and dampening of the interaction between TBK1/IKKepsilon and DDX3. J. Gen. Virol..

[10] Cheng J., Imanishi H., Morisaki H., Liu W., Nakamura H., Morisaki T., Hada T. (2005). Recombinant HBsAg inhibits LPS-induced COX-2 expression and IL-18 production by interfering with the NFkappaB pathway in a human monocytic cell line, THP-1. J. Hepatol..

[11] Vincent I.E., Zannetti C., Lucifora J., Norder H., Protzer U., Hainaut P., Zoulim F., Tommasino M., Trépo C., Hasan U. (2011). Hepatitis B virus impairs TLR9 expression and function in plasmacytoid dendritic cells. PLoS One.

[12] Xu Y., Hu Y., Shi B., Zhang X., Wang J., Zhang Z., Shen F., Zhang Q., Sun S., Yuan Z. (2009). HBsAg inhibits TLR9-mediated activation and IFN-alpha production in plasmacytoid dendritic cells. Mol. Immunol..

[13] Kumar M., Jung S.Y., Hodgson A.J., Madden C.R., Qin J., Slagle B.L. (2011). Hepatitis B virus regulatory HBx protein binds to adaptor protein IPS-1 and inhibits the activation of beta interferon. J. Virol..

[14] Wei C., Ni C., Song T., Liu Y., Yang X., Zheng Z., Jia Y., Yuan Y., Guan K., Xu Y. (2010). The hepatitis B virus X protein disrupts innate immunity by downregulating mitochondrial antiviral signaling protein. J. Immunol..

[15] Liu Y., Li J., Chen J., Li Y., Wang W., Du X., Song W., Zhang W., Lin L., Yuan Z. (2015). Hepatitis B virus polymerase disrupts K63-linked ubiquitination of STING to block innate cytosolic DNA-sensing pathways. J. Virol..

[16] Wang H., Ryu W.S. (2010). Hepatitis B virus polymerase blocks pattern recognition receptor signaling via interaction with DDX3: Implications for immune evasion. PLoS Pathog..

[17] Yang P.L., Althage A., Chung J., Chisari F.V. (2002). Hydrodynamic injection of viral DNA: A mouse model of acute hepatitis B virus infection. Proc. Natl. Acad. Sci. USA.

[18] Lin Y.J., Huang L.Y., Yang H.C., Tzeng H.T., Hsu P.N., Wu H.L., Chen P.J., Chen D.S. (2010). Hepatitis B virus core antigen determines viral persistence in a C57BL/6 mouse model. Proc. Natl. Acad. Sci. USA.

[19] Leong C.R., Oshiumi H., Okamoto M., Azuma M., Takaki H., Matsumoto M., Chayama K., Seya T. (2015). A MAVS/TICAM-1-independent interferon-inducing pathway contributes to regulation of hepatitis B virus replication in the mouse hydrodynamic injection model. J. Innate Immun..

[20] Burdette D.L., Vance R.E. (2013). STING and the innate immune response to nucleic acids in the cytosol. Nat. Immunol..

[21] Gao P., Ascano M., Zillinger T., Wang W., Dai P., Serganov A.A., Gaffney B.L., Shuman S., Jones R.A., Deng L. (2013). Structure-function analysis of STING activation by c[G(2',5')pA(3',5')p] and targeting by antiviral DMXAA. Cell.

[22] Guo F., Han Y., Zhao X., Wang J., Liu F., Xu C., Wei L., Jiang D.J., Block T.M., Guo J.T. (2015). STING Agonists Induce an Innate Antiviral Immune Response against Hepatitis B Virus. Antimicrob. Agents Chemother..

[23] Grimm D., Thimme R., Blum H.E. (2011). HBV life cycle and novel drug targets. Hepatol. Int..

[24] Burdette D.L., Monroe K.M., Sotelo-Troha K., Iwig J.S., Eckert B., Hyodo M., Hayakawa Y., Vance R.E. (2011). STING is a direct innate immune sensor of cyclic di-GMP. Nature.

[25] Suzuki T., Oshiumi H., Miyashita M., Aly H.H., Matsumoto M., Seya T. (2013). Cell type-specific subcellular localization of phospho-TBK1 in response to cytoplasmic viral DNA. PLoS One.

[26] Shimoda S., Harada K., Niiro H., Shirabe K., Taketomi A., Maehara Y., Tsuneyama K., Nakanuma Y., Leung P., Ansari A.A. (2011). Interaction between Toll-like receptors and natural killer cells in the destruction of bile ducts in primary biliary cirrhosis. Hepatology.

[27] Hösel M., Quasdorff M., Wiegmann K., Webb D., Zedler U., Broxtermann M., Tedjokusumo R., Esser K., Arzberger S., Kirschning C.J. (2009). Not interferon, but interleukin-6 controls early gene expression in hepatitis B virus infection. Hepatology.

[28] Aravalli R.N. (2013). Role of innate immunity in the development of hepatocellular carcinoma. World J. Gastroenterol..

[29] Ganem D., Prince A.M. (2004). Hepatitis B virus infection—Natural history and clinical consequences. N. Engl. J. Med..

[30] Seeger C., Mason W.S. (2000). Hepatitis B virus biology. Microbiol. Mol. Biol. Rev..

[31] Sato S., Li K., Kameyama T., Hayashi T., Ishida Y., Murakami S., Watanabe T., Iijima S., Sakurai Y., Watashi K. (2015). The RNA sensor RIG-I dually functions as an innate sensor and direct antiviral factor for hepatitis B virus. Immunity.

